# Type of Treatment Supporters in Successful Completion of Tuberculosis Treatment: A Retrospective Cohort Study in Pakistan

**DOI:** 10.2174/1874279301810010037

**Published:** 2018-05-18

**Authors:** Sana Hussain, Jamshed Hasnain, Zareen Hussain, Masroor Badshah, Hafeez Siddique, Christina Fiske, April Pettit

**Affiliations:** 1 Shaheed Zulfiqar Ali Bhutto Institute of Science and Technology, (SZABIST), Karachi, Pakistan; 2 Bridge Consultants Foundation, Karachi, Pakistan; 3 Ziauddin University, Karachi, Pakistan; 4 Northwest School of Medicine, Peshawar, Pakistan; 5 Vanderbilt University, Nashville, TN, USA

**Keywords:** Sindh, Treatment outcome, TB, Family member, TB DOTs, Treatment Supporters

## Abstract

**Background:**

The World Health Organization has recommended a patient-centered approach to tuberculosis drug administration. A central element of the patient-centered strategy is the use of treatment supporters to evaluate and elevate adherence to the treatment regimen and to address poor adherence when it occurs. This study was led to determine the part of various treatment supporters in the successful completion of treatment.

**Method:**

This study was conducted in two locales of Sindh, Hyderabad and Mirpurkhas. Information gathered included age, gender, regions, sort of treatment supporters (relatives, community and health facility workers) and treatment outcomes.

**Results:**

Of the 773 patients incorporated into the study, 86.8% picked a family supporter, 7.63% selected community worker and 5.56% chose health facility worker as their treatment supporter. Women and younger patients were more likely to prefer that family members supervise their treatment. Treatment achievement rates among the patients regulated by the three kinds of treatment supporters, were not altogether unique in relation to each other (*p*=0.23 Chi square).

**Conclusion:**

The study demonstrates that TB patients ought to be urged to pick the supporter of their inclination as selection of treatment supporter outside the health system does not adversely affect TB treatment outcomes in limited resource settings.

## 1. INTRODUCTION

Tuberculosis is thought to be a standout amongst the most universally deadliest and transmissible infections. An expected 10.4 million individuals fell sick with TB in 2016 and 56% were in five nations: India, Indonesia, China, the Philippines and Pakistan [1]. Tuberculosis (TB) is a major cause of morbidity and mortality in Pakistan. In 2016, the treatment success rate with new cases was 93% in Pakistan [1], but these statistics need to be improved in Sindh so that the Millennium Development Goals for TB can be achieved and incidence of Multidrug Resistance Tuberculosis may be reduced in the province.

The World Health Organization (WHO) advocates Directly Observed Therapy (DOT) for TB treatment, in which it is ensured that the patient is taking medicines as observed by a treatment supporter [2]. These treatment supporters play a vital role in guiding and motivating TB patients thus ensuring completion of treatment. Treatment supporters can be a facility-based health worker, a community-based health worker or a family member. Previously, WHO treatment guidelines did not support the idea of a family member playing the role of a treatment supporter because most family members are not medically trained and may not be empowered to successfully impact whether a patient takes their TB medications, thus increasing the chances of treatment interruption [3]. Latest guidelines have conditionally recommended community or home-based Directly Observed Treatment (DOT) over health facility-based DOT or unsupervised treatment [4].

In Sindh, Pakistan, finding a facility-based health worker or a community-based health worker to act as a DOT treatment supporter can be challenging, as the number of these trained health workers is low and patients may not feel comfortable accepting these supporters because of cultural and social reasons.

The province Sindh has an estimated populace of 42.4 million people, roughly equal rural (51.2%) and urban (48.8%) inhabitants. Sindh territory has 23 regions [5]. Sindh is one cosmopolitan area in Pakistan and is characterized by a wide gap between rich and poor people with unequal access to health care services. Occupants of low-income neighborhoods, experience the ill effects of overcrowding and malnutrition. Therefore, they are susceptible to developing tuberculosis [6]. Investigations of TB directed in various districts of the Sindh region have generally relied on a little populace, old writing and the Pakistan’s national TB program (NTP) data [7]. There is little data on TB treatment outcomes in relation to the type of treatment supporter in Sindh. We therefore conducted this study in Hyderabad and Mirpurkhas, the two major districts of Sindh to determine the proportion of TB patients opting for a family member as their treatment supporter and to describe the association of different types of treatment supporter with the TB treatment success rates.

## 2. METHODS

We conducted a retrospective cohort study to examine the association of different categories of treatment supporters with treatment outcomes of TB patients registered from 2009–2013 under the National TB control program (NTP) at two TB clinics in Hyderabad and Mirpurkhas, two of the major districts of the Sindh province.

Patients with TB who opted for a treatment supporter and whose treatment outcome was documented in the standard TB register maintained at each site, were included in the study. Information obtained from chart review included, age, gender, district, type of treatment supporter and treatment outcomes. Treatment outcomes were defined as per WHO guidelines: 1) cured meant a patient who was initially smear-positive and who was smear-negative in the last month of treatment and on at least one previous occasion; 2) treatment completed meant a smear positive patient who has completed the duration of treatment and has at least one follow up smear negative results but none at the end of treatment due to any reason; 3) treatment failure indicated a patient who was initially smear-positive and who remained smear-positive at month 5 or later during treatment; 4) default which occurred if a patient interrupted treatment for at least 2 months after initiation of treatment [8]. It was also noted if a patient died from any cause during treatment or if the patient transferred to another reporting unit and for whom the treatment outcome was not known. The current success rate was defined as the sum of cured patients and patients who completed and expressed as percentage.

## 3. STATISTICAL ANALYSIS

Data were entered twice by two separate computer operators using Microsoft Access (Microsoft Corporation, New Mexico, US). Summary statistics were calculated for continuous variables and frequencies were computed for categorical variables. *X^2^* was used to compare different variables among the three treatment support groups. A p value of <0.05 was considered significant. Data were analyzed on Statistical Package for Social Sciences (SPSS) V.20.0 (IBM Corporation).

## 4. RESULTS

There were 1249 eligible patients during the study period, of which 773 patients met the inclusion criteria. During the study period, 617 successfully completed treatment, 87 defaulted, 38 died, 9 had treatment failure and 22 patients were transferred out.

Six hundred and seventy-one patients (86.8%) chose a family member as their treatment supporter, 59 (7.63%) selected a community-based health worker and 43 (5.56%) chose a facility- based health worker. Demographic characteristics of these patients in relation to their DOT supporters are given in Table 1. While, TB treatment outcomes in relation to the type of treatment supporter are given in Table 2.

Table 1 shows the baseline characteristics of patients with tuberculosis in relation to the type of treatment supporters. The result shows that family members were the most preferred type of treatment supporters opted by the patients and 86.8% patients selected family members as their treatment supporters. Of these patients, 39.9% males and 60% females selected family members as their treatment supporters. Further, it has been found that women and younger patients preferred family members as their treatment supporters.

Table 2 shows the result of chi square analysis. Result indicates that treatment success rates of the TB patients supervised by the three types of DOT supporter, were not significantly different from each other (p=0.23). The treatment success rate was 80.4% among the patients who selected family members as their treatment supporter, 79.06% treatment success rate was among the patients who opted health facility member as their treatment supporter while, 73% success rate was found among the patients whose treatment supporters were from the community.

## 4. DISCUSSION

This study exhibits that the majority of TB patients in the target districts favored a relative over a community-based health worker or a facility-based health worker, as their treatment supporter, and the TB treatment outcomes were not affected by the type of treatment supporter picked.

Young females were especially disposed to choose a family member as a treatment supporter. This result is in accordance with the discovery from a randomized control trial led in two other districts of Sindh *i.e.* Karachi and Umerkot. The result of the study ascertains that 33% males and 66% of female TB patients favored a relative as their treatment supporter, and the treatment results were not influenced by the kind of treatment supporter chosen [9]. Results from other monetarily and socially comparative nations are likewise in concurrence with these findings. A retrospective study in Thailand found that 90% of patients selected a family member [10]. Similarly, a cluster-randomized trial in Nepal showed that 89% of patients opted for a family person, while a retrospective cohort study in Zimbabwe found this figure to be 40%, second highest among the four treatment supporter categories [11, 12]. A large community randomized trial in South Africa showed that 59% of the TB patients who could choose their treatment supporter would opt for a family member [13].

This is justifiable in Pakistan (i) for social and cultural reasons the majority of TB patients in Sindh want to be regulated by a nearby relative, (ii) since there is a lot of stigma and discrimination related with this infection, TB patients, particularly females, do not want to be seen by a pariah in light of dread of being unveiled as a TB case in the community, and (iii) people in Pakistan by and large don’t effectively trust people from different groups going to their living arrangements routinely. These components could clarify why a relative was picked by most by far of patients as their treatment supporter.

This study also showed that treatment success rate of the TB patients who were supervised by a family member (80%) is comparable to the success rate observed among patients overseen by facility- based health workers (79%) and community-based health workers (73%). These findings closely match the results obtained by several other researchers. A randomized control trial conducted in northern Pakistan concluded that there was no significant difference in the treatment success rates of patients supervised by a family member (62%), health facility based treatment supporter (67%) and self- administration (62%) [14]. A cohort analysis in Tanzania revealed similar results in which success rates were not significantly different between the control group and patient centered treatment group (70% Vs 82%). In this study patients selected their own treatment supporter. Ninety-four percent of patients opted for a family member, and the authors concluded that family members acting as treatment supporters was not likely to lead to adverse TB treatment outcomes and was acceptable [15]. Likewise a randomized trial from Swaziland reported that success rates of the patients observed by community based health worker and a family based DOT supporter were very similar (66% Vs 68%) [16]. Comparable results were also reported from studies in Malawi and Australia [17, 18].

Although the results of this study support the idea of promoting family members as DOT supporters the WHO has advocated only selecting, such an individuals if all other options have been exhausted for several reasons. Family members are usually not medically trained and may not be able to identify adverse effects of TB medications. Further, because of cultural and social reasons, the family member may not be empowered to enforce the strict adherence to TB treatment that is required for cure. However, in a resource limited country like Pakistan it is difficult to find sufficient numbers of trained facility- based or community-based health workers. Family members can thus play a vital role as DOT supporters particularly in the less developed and remote areas of Sindh province.

This study had several limitations. We only collected data from two districts in Sindh and therefore we cannot assume the results are representative of the entire province. However these districts are demographically similar to rest of the province. Also, we did not have demographic information on the treatment supporters themselves.

## CONCLUSION

In conclusion, this study demonstrates that TB treatment outcomes are not affected by the treatment supporter chosen to supervise DOT. In resource limited settings, to satisfy society’s obligations and to care for individual patients effectively, it is essential that patients should be allowed to choose any supporter with whom they feel comfortable. Further, each culture is unique and has particular strengths and weaknesses. In resource limited countries, before implementing direct observation of treatment, it is imperative to identify and enlist the cultural related strengths and flaws, then support needs to be scaled up for TB treatment supervision whether, outside the health system or within the health system.

## Figures and Tables

**Table 1:** Baseline characteristics of patients with tuberculosis in relation to types of DOT in Mirpurkhas and Hyderabad districts of Sindh Pakistan

Category		Type of Treatment Supporters		
Sex	Total	Health Care Provider Male/Female	Community Health Worker Male/Female	Family Member Male/Female
Male	326	21 (48.8%)	37 (62.7%)	268 (39.9%)
Female	447	22 (51.1%)	22 (37.2%)	403 (60%)
**Total**	**773**	**43 (5.56%)**	**59 (7.63%)**	**671 (86.8%)**
**Age Group**	–	–	–	–
1–15		0/0	1/1	13/26
**16–25**		**8/6**	**11/8**	**99/188**
26–35		1/6	6/7	31/75
36–45		3/5	6/3	23/55
46–55		6/2	5/3	53/34
Above 55		3/3	8/0	49/25

**Table 2:** TB treatment outcomes in relation to different types of DOT supporter in Mirpurkhas and Hyderabad districts of Sindh Province Pakistan

Category	Family Supporter	Health Facility Supporter	Community Supporter	Statistical Indicator

**Starting Treatment**	671	43	59	χ2 = 12.86 df =10 p =0.23
**n (%)**	(86.8%)	(5.56%)	(7.63%)	

**Treatment Success**	**540**	**34**	**43**	
**n (%)**	**(80.4%)**	**(79.06%)**	**(73%)**	

**Defaulted**	72	4	11	
**n (%)**	(10.7%)	(9.3%)	(18.64%)	

**Failure**	6	2	1	
**n (%)**	(0.89%)	(4.65%)	(1.64%)	

**Died**	35	1	2	
**n (%)**	(5.2%)	(2.32%)	(3.38%)	

**Transferred Out**	18	2	2	
**n (%)**	(2.68%)	(4.65%)	(3.38%)	

**Total**	**671**	**43**	**59**	**773**
